# Machine learning models’ assessment: trust and performance

**DOI:** 10.1007/s11517-024-03145-5

**Published:** 2024-06-08

**Authors:** S. Sousa, S. Paredes, T. Rocha, J. Henriques, J. Sousa, L. Gonçalves

**Affiliations:** 1 CISUC, Center for Informatics and Systems of University of Coimbra, University of Coimbra, Pólo II, 3030-290 Coimbra, Portugal; 2 Polytechnic Institute of Coimbra, Coimbra Institute of Engineering (IPC/ISEC), Rua Pedro Nunes, 3030-199 Coimbra, Portugal; 3 Department of Cardiology, Instituto Português de Oncologia do Porto Francisco Gentil, E.P.E., Porto, Portugal; 4 Cardiology Department, Centro Hospitalar e Universitário de Coimbra, Praceta Professor Mota Pinto, 3004-561 Coimbra, Portugal

**Keywords:** Trust, Interpretability, Explainable AI, Clinical decision support systems

## Abstract

**Abstract:**

The common black box nature of machine learning models is an obstacle to their application in health care context. Their widespread application is limited by a significant “lack of trust.” So, the main goal of this work is the development of an evaluation approach that can assess, simultaneously, trust and performance. Trust assessment is based on (i) model robustness (stability assessment), (ii) confidence (95% CI of geometric mean), and (iii) interpretability (comparison of respective features ranking with clinical evidence). Performance is assessed through geometric mean. For validation, in patients’ stratification in cardiovascular risk assessment, a Portuguese dataset (*N*=1544) was applied. Five different models were compared: (i) GRACE score, the most common risk assessment tool in Portugal for patients with acute coronary syndrome; (ii) logistic regression; (iii) Naïve Bayes; (iv) decision trees; and (v) rule-based approach, previously developed by this team. The obtained results confirm that the simultaneous assessment of trust and performance can be successfully implemented. The rule-based approach seems to have potential for clinical application. It provides a high level of trust in the respective operation while outperformed the GRACE model’s performance, enhancing the required physicians’ acceptance. This may increase the possibility to effectively aid the clinical decision.

**Graphical abstract:**

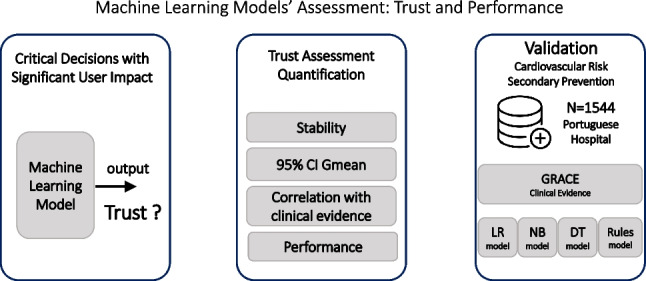

## Introduction

The application of machine learning (ML) models to critical areas, involving decisions with significant user impact, faces additional challenges. In this context, the European Union General Data Protection Regulation recently approved the “right to explanation,” i.e., the right to information about individual decisions made by algorithms [1]. This increasing demand for interpretable high-performing machine learning models in real-world applications has given rise to the field of explainable artificial intelligence (XAI). It focuses on developing models that are not only accurate but also transparent and comprehensible to humans, enabling users to understand and interpret the behavior of classification models [2, 3]. In opposition to the traditional lack of interpretability of ML models, since they often behave as “black-box” systems, this new perspective is decisive for improving the trust and consequent adoption of ML models in critical applications [2].

There are several fields where ML algorithms have achieved remarkable performances, which is the case of risk assessment models [4, 5]. Despite this evidence, the mentioned lack of interpretability is an unavoidable barrier to their widespread application, as it does not provide trust in the ML model operation. Actually, whenever there is a significant and direct impact on the user’s life, e.g., healthcare context, it is not possible to conjugate a black box model with the required trust for its application in practice.

The main objective of this work is the development of an agnostic assessment scheme that can be effective in the simultaneous evaluation of trust and performance of a ML model. Thus, the proposed methodology is based on these two different perspectives. Trust assessment considered three different perspectives: (i) model robustness based on stability assessment; (ii) confidence, by the computation of the 95% confidence interval of geometric mean; and (iii) interpretability, through the generation of a features ranking and its comparison with clinical evidence-based feature importance. Performance evaluation was assured through the computation of the geometric mean (*G*_*mean*_). The innovation of this work is precisely the quantification, based on different objective metrics, of trust in the operation of an ML model.

This global assessment can be considered in different contexts. In this work, it was applied to cardiovascular disease (CVD), as a proper patients’ risk stratification allows the optimization of personalized healthcare plans. This support to clinical decision may assume an enormous importance, as CVD are the leading cause of morbidity and mortality in the world, e.g., 17.9 million deaths only in 2019 [6]. Currently, GRACE score is the most applied CVD risk assessment tool in the Portuguese daily clinical practice [7]. It is specific to acute coronary syndrome (ACS) patients (secondary prevention), and it is included in the clinical guidelines. So, in this work, it is assumed as the clinical reference, i.e., it expresses the current clinical evidence.

The proposed assessment is validated through the comparison between some ML models (white-box models) and GRACE score (clinical reference).

Some main phases can be identified: (i) implementation of interpretable ML models to evaluate the 6-month mortality risk of ACS patients after hospital admission; (ii) identification of metrics to quantify the trust of the different models; and (iii) comparison of ML models with GRACE regarding both performance and trust. This CVD use case is supported by a Portuguese dataset provided by the Coimbra Hospital and Universitary Centre (CHUC) comprising *N*=1544 acute coronary syndrome (ACS) patients admitted to CHUC.

## Methods

This proposed global assessment comprises the combination of two different perspectives: trust and performance.

### Trust evaluation

Adapting the taxonomy adopted in [8], besides the proposed quantitative assessment of trust (commonly designated as functionally grounded evaluation), a qualitative assessment performed by domain experts can also be accomplished (commonly designated as application-grounded evaluation).

The functionally grounded evaluation is based on properties. However, despite some recent research in this area [9–11], there are few quantitative metrics available in literature [9]. As mentioned, this work addresses this type of evaluation based on three different concepts: (i) model robustness; (ii) confidence; and (iii) interpretability.

#### Model robustness

The model robustness is based on stability evaluation, that has been defined by several authors [8, 12–14], as the ability of a model to consistently assign the same label to similar instances. In other words, a stable model should produce similar outputs for similar inputs, providing reliable and predictable results.

In order to evaluate if models compute the same label to similar instances, the stability measure (1) was implemented based on [14].

1 $${S}_t\left(\boldsymbol{x}|{S}^{+},{S}^{-}\right)=\frac{\sum_{{\boldsymbol{x}}_{\boldsymbol{i}}\epsilon {S}^{+}}e\left(-\frac{{\left\Vert \boldsymbol{x}-{\boldsymbol{x}}_i\right\Vert}^2}{2{\sigma}^2}\right)-{\sum}_{{\boldsymbol{x}}_{\boldsymbol{j}}\epsilon {S}^{-}}e\left(-\frac{{\left\Vert \boldsymbol{x}-{\boldsymbol{x}}_j\right\Vert}^2}{2{\sigma}^2}\right)}{k}$$

where:

- **x** *ϵR*^*n*^ is a given instance for which the stability is assessed.
- *S*^+^ is the set of neighbors with the same output than **x**.
- *S*^−^ is the set of neighbors with the opposite output to **x**.
- *k* is the number of neighbors of **x**.
- *σ* is the standard deviation in *S* $\left(\sigma =\frac{1}{k}{\sum}_{{\textbf{x}}_{\textbf{i}}\epsilon S}\left\Vert \textbf{x}-{\textbf{x}}_{\textbf{i}}\right\Vert \right)$.

Stability measure is in the range [−1,1]: −1 if all the *k* neighbors belong to a class different from the class of **x**, while value 1 means that all the *k* neighbors belong to the same class as **x**. If *k* is an even number, where half of the neighbors belong to a different class and the other half belongs to the same class as **x**, then the stability value is 0. The global stability of each model corresponds to the average of the stability obtained for all individual patients in the dataset. Contrarily to [14], in (1) all the neighbors of a given instance were considered.

#### Confidence—95% CI of the ***G***_***mean***_

The evaluation of stability, without the assessment of the respective performance, could be misleading, e.g., a poor model that has the same label to all patients achieves a very high stability. Therefore, this evaluation should be combined with a confidence measure, assuring that minor changes to the input data only lead to slight changes in the output. The narrower the range of CI, the more confidence in the measure since it is more precise. In this work, the 95% CI on the *G*_*mean*_ was computed to complement the results provided by the stability assessment. Those intervals were obtained through the non-parametric leave-one out bootstrap method.

#### Interpretability

As stated in [13, 15], the quantification of the quality of explanations is not obvious neither consensual. Many authors underline the difficulty in this measurement, e.g., in [13], the author argues that this issue is generally very challenging, while in [15, 16], authors state that there is no way of knowing “how correct an explanation is.” Although, in [17] is proposed: “that the concordance in explanations as well as how well the explanations align with what is already known in the domain will determine the explanation preference.” This work follows this approach, intending to measure that alignment through the determination of features rank based on Shapley values [18]. The goal is the generation of a features ranking and its posterior comparison with clinical evidence-based feature importance. This comparison was performed based on correlation between ranks.

**Fig. 1:**
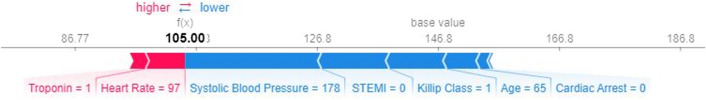
SHAP force plot (GRACE score)

There are some software tools dedicated to the visualization of Shapley values. Those values can be visualized as *forces* in a force plot (Fig. 1). The Shapley value for each feature is a force that pushes to increase (positive Shapley value) or decrease (negative Shapley value) the prediction for a specific data instance. The interpretation of the Shapley value for a feature value is the contribution to the prediction for this particular instance, compared to the average prediction for the dataset. In this plot, the base value is also represented and consists of the average of all predictions. Furthermore, different implementations of SHAP allow alternative visualizations based on the aggregation of Shapley values.

### Performance evaluation

The performance assessment of a classification problem can be addressed through the simultaneous consideration of sensitivity (SE) and specificity (SP) (2):

2 $$SE=\frac{TP}{TP+ FN};\;SP=\frac{TN}{TN+ FP}$$

where TP is a true positive instance, TN is a true negative instance, FN is an instance incorrectly predicted as negative, while FP is an instance incorrectly predicted as positive. To assure the simultaneous maximization of these metrics, the geometric mean (3) was considered.

3 $${G}_{mean}=\sqrt{SE\times SP}$$

To compare the performance between the GRACE and ML models, considering the geometric mean values obtained across 10 different runs, the Mann-Whitney *U* statistical test was applied for each pair of methods. The goal was to determine if we could reject the null hypothesis that assumes that the median of the *G*_*mean*_ is the same across each pair of methods. To provide a visual representation of the results of the several methods applied, boxplots of the results of the 10 runs across the different methods were also implemented.

### Use case: cardiovascular risk assessment

**Fig. 2:**
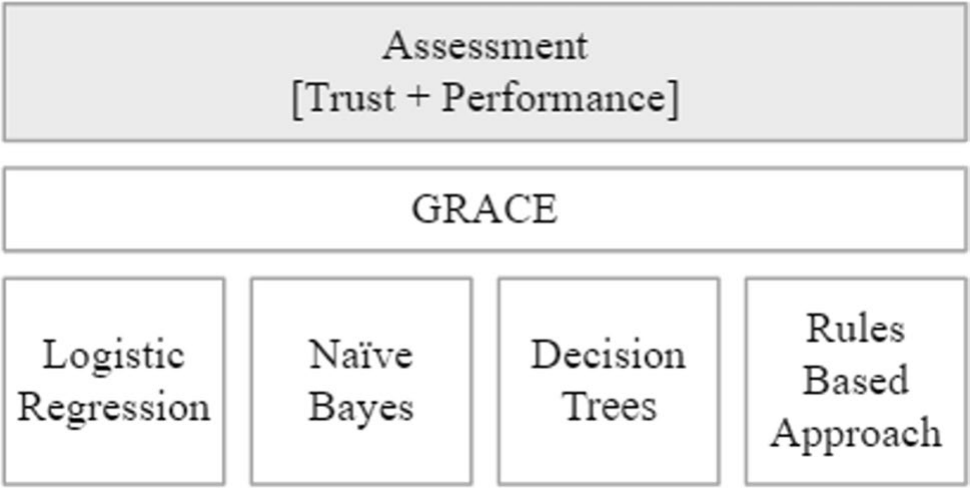
Global assessment applied to CVD risk assessment

The proposed global assessment was applied to cardiovascular risk assessment context (Fig. 2). The main objective was to globally compare four ML models with GRACE score, as it is the clinical reference, i.e., the risk assessment tool specific to acute coronary syndrome patients recommended by clinical guidelines [7].

#### GRACE risk score: clinical reference

The GRACE risk score was developed for a short-term risk assessment (6 months; myocardial infarction (MI)/death), after hospital admission with ACS diagnosis. It was supported on an international registry of patients across the entire spectrum of ACS (MI STEMI/NSTEMI and unstable angina) [7]. In the GRACE risk score, the final score of a patient is the sum of all variables’ scores (ranges from 2 to 383). Each variable score is attributed according to Table 1 .

**Table 1:** GRACE risk score

Variable	Range	Score	Variable	Range	Score
Age (years)	< 40 40–49 50–59 60–69 70–79 > 80	0 18 36 55 73 91	Creatinine (mg/dl)	0–0.39 0.4–0.79 0.8–1.19 1.2–1.59 1.6–1.99 2–3.99 >4	2 5 8 11 14 23 31
Heart rate (bpm)	<70 70–89 90–109 110–149 150–199 >200	0 7 13 23 36 46	Killip class	I II III IV	0 21 43 64
Systolic blood pressure (mmHg)	<80 80–99 100–119 120–139 140–159 160–199 >200	63 58 47 37 26 11 0	Elevated cardiac markers	No Yes	0 15
Cardiac arrest at admission	No Yes	0 43	ST-segment deviation	No Yes	0 30

The original GRACE provides three levels of patient risk (low, intermediate, high). However, mapping for a binary classification problem is often required. Usually, the option that originates better results is 0 for low risk or intermediate risk, while 1 denotes a high risk patient. This perspective was confirmed by the physicians that collaborated in this work. GRACE is included in current clinical guidelines and is the most applied risk score in Portugal [7]. As represented in Fig. 2, it was identified as the clinical reference, so it was globally compared with the four selected white-box ML models.

#### Logistic regression

Logistic regression (LR) is, typically, applied in binary classification problems. It computes the probabilities based on the logistic function:

4 $$P\left({y}^{(i)}=1\right)=\frac{1}{1+\exp \left(-\left({\beta}_0+{\beta}_1{x}_1^{(i)}+\dots +{\beta}_p{x}_p^{(i)}\right)\right)}$$

The probability is given by (4), where *i* represents a specific instance, *p* the number of features, *x*_*i*_ the different features, and *β*_*i*_ the learned feature weights/coefficients. Considering a decision threshold and the estimated probability, a given instance is classified as positive or negative (high/low risk). Odds is an important concept in LR models, they are given by the probability of an *event* divided by the probability of *no event* and relate with the regression coefficients through (5):

5 $$\frac{P\left(y=1\right)}{1-P\left(y=1\right)}= odds=\exp \left(-\left({\beta}_0+{\beta}_1{x}_1^{(i)}+\dots +{\beta}_p{x}_p^{(i)}\right)\right)$$

The *odds ratio* (6) is the ratio between two odds, e.g., the odds when a numerical feature is changed by one unit divided the odds when the numerical feature remains unchanged.

6 $$\frac{odds_{x_j+1}}{odds}=\exp \left({\beta}_j\right)$$

Through odds ratio is possible to determine how likely a given feature may influence a specific event [19]. According to (6), if the coefficient is positive (negative), the change in the odds ratio will be higher (lower) than 1 [16]. In order to allow a direct comparison among odds ratio for continuous variables, the numerical values must be normalized for the same scale. After normalization, e.g., *z*-score normalization, the standardized coefficients can be directly compared about the respective relative importance to the outcome. Binary variables can also be directly compared among them, as they assume the same values, providing some insights about the respective feature importance. Therefore, the interpretability of a logistic regression model is addressed based on the coefficients and in the respective odds ratio.

#### Naïve bayes

**Fig. 3:**
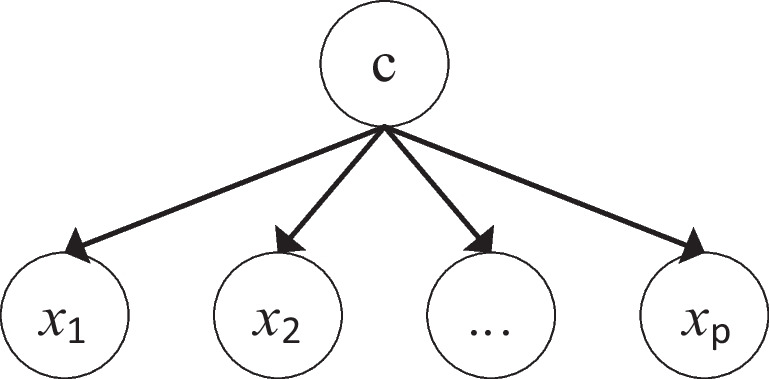
Naïve Bayes structure

The structure of Naïve Bayes (NB) (Fig. 3) is particularly well adapted to clinical problems:

*x*_*i*_ is an observation (e.g., clinical exam) and c a hypothesis (e.g., have a disease). The term P(c| *x*_*i*_) denotes a posterior probability, i.e., the probability of the hypothesis after having seen the observation *x*_*i*_ (e.g., probability to have a disease given the results of a clinical exam). P(c) is the probability of the hypothesis before seeing any observation (e.g., prevalence of the disease). P(*x*_*i*_| c) is a likelihood, the probability of the observation if the hypothesis is true (e.g., sensitivity of the clinical exam). The final classification *c* is achieved based on (7):

7 $$c= argmax\left(\alpha P\left({c}_j\right)\prod_{i=1}^pP\left({x}_i|{c}_j\right)\right)$$

where *c*_*j*_ is a mutually exclusive class of *c*, *x*_*i*_ is the value of an attribute that belongs to the instance **x** = [*x*_1_, …, *x*_*p*_], and *α* is a normalization constant.

The inference mechanism of this algorithm relies on conditional probabilities, i.e., *P*(*x*_*i*_| *c*_*j*_). This refers to the probability of each category within each feature (*x*_*i*_), given a particular class *c*_*j*_. The model has to learn from the training dataset, these conditional probabilities as well as the prior probabilities [20]. The assessment of these probabilities allows some level of model’s interpretability.

#### Decision trees

This well-known algorithm is based on learning simple decision rules directly inferred from data. There are several algorithms to build decision tree (DT), namely classification and regression trees (CART), iterative dichotomizer 3 (ID3), and C4.5. The CART algorithm takes a feature and determines which cut-off point minimizes the Gini index (8):

8 $${G}_i=1-\sum\limits_{k=1}^n{p}_{i,k}^2$$

where *p*_*i*, *k*_ is the ratio of *k* instances among the training instances in the *i*^*th*^ node considering the *n* classes. The Gini index is an impurity metric, i.e., instances in a node have different class values, so it should be as low as possible. After the determination of the best cutoff for each feature, the algorithm selects the feature for splitting that would result in the best partition (lowest value of Gini index). This split is added to the tree, creating different subsets of the dataset. The algorithm continues recursively until a stop criterion is reached, e.g., the minimum number of samples required to split an internal node [16]. Decision rules are inherently interpretable. Feature importance can be directly derived from the DT structure, given their visualization property. However, DT have the important disadvantage of being unstable, i.e., small variations in the data may change the entire tree structure, which may affect the performance and the confidence in the model [16].

#### Rule-based approach

**Fig. 4:**
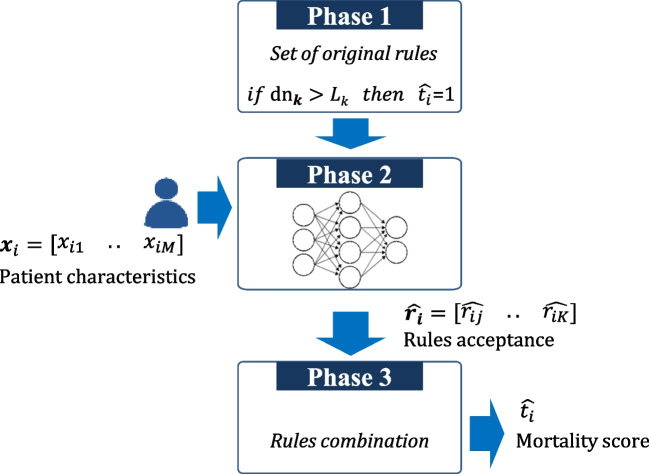
Rule-based approach [21]

This approach (Fig. 4) was previously developed by this research team [5, 21], it is composed of three main phases:

1. Creation of a set of interpretable rules, based on clinical evidence, describing the problem under analysis.
2. Application of a ML model to identify the more appropriated subset of rules for each patient, according to their particular characteristics.
3. Estimation of each patient cardiovascular mortality risk based on the selection of a subset of rules from the original set.

This strategy intends to simulate the physician’s reasoning: (1) based on clinical knowledge (set of rules); (2) to address the specific characteristics of a patient (personalized choice of rules); and (3) the physician applies only the most suitable rules to achieve a diagnosis.

##### Derivation of rules

In its simplest form, a rule is a simple binary association based on one risk factor, as described in the following example (9):

9 $$if\ Killip=4\ then\ \hat{t}=1$$

where $\hat{t}$ is the estimated outcome {0, survival; 1, death}. More complex rules, combining two or more risk factors, might also be considered. The set of baseline rules was directly derived from available data and afterwards validated by the clinical partner. In order to promote direct interpretability and acceptance, it is critical that rules not only reflect the data distribution but also be aligned with the clinical guidelines. The rules creation process is fully detailed in [21].

##### Selection of correct rules

In clinical practice, a physician identifies a subset of rules to perform a diagnosis, considering each patient’s specific characteristics. This step intends to simulate this logic, by selecting the most likely subset of rules suitable for a given patient.

**Fig. 5:**
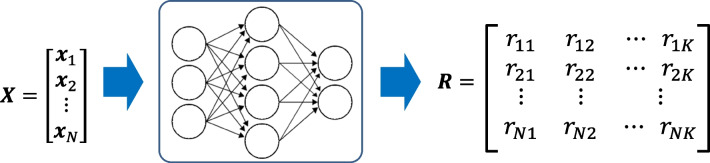
Training ML model to address the correctness of rules [21]

Figure 5 represents the implemented scheme. The initial set of rules is applied to all patients in order to determine the correctness of each rule for each patient. Then, a ML model is created to estimate the correctness of rules for a new unseen instance.

The target, i.e., the correctness of each rule can be expressed as 0, *incorrect*; 1, *correct* since the patient’s outcome, and the output of each rule are known.

For example, considering the rule (10):

10 $$if\ albumin\le 25\ then\ \hat{t}=1$$

if a patient with an albumin of 30 dies, then this rule is not correct for this patient and should not be applied. A ML model can be created, where the input is the features (risk factors) matrix (*N* patients), ***X****ϵR*^*N*, *M*^, the output (target) is a matrix ***R****ϵR*^*N*, *K*^. Each value *r*_*ik*_ provides an estimation of the correctness of the rule *k* for the patient *i*, a binary value {0,1}. A value of *r*_*ik*_=1 (rule correct) means that the rule *k* should be accepted and combined to assess the mortality risk of the patient *i*. In contrast, a value of *r*_*ik*_=0 (rule incorrect) means that rule *k* should not be used in the assessment of the mortality risk for patient *i*.

A new patient is submitted to this model and the respective output is a vector that contains the correctness of different rules. Only the rules identified as correct are applied in the final classification (next step).

##### Estimation of individual risk

The estimation of the mortality patient risk is exclusively based on the subset of selected rules. In the simplest case, a majority voting can be implemented, i.e., the risk level is the most common output of the accepted rules. Additionally, an estimation for the mortality patients risk based is also introduced on Equation (11).

11 $${\hat{t}}_i=\frac{1}{Q}\sum_{j=1}^Q{\hat{r}}_{ij}\bullet {\hat{t}}_{ij}$$

*Q* is the subset of accepted rules, i.e., those rules that verify ${\hat{r}}_{ik}=1$. The patient mortality score is calculated through the ratio between the number of accepted rules that suggest mortality (${\hat{r}}_{ik}=1\wedge {\hat{t}}_{ik}=1$) and the number of all accepted rules (*Q*). This ${\hat{t}}_i$ score relates directly with the final prediction as defined in (12).

12 $${\hat{t}}_i=\left\{\begin{array}{cc}1\ (death)& if\ {\hat{t}}_i\ge 0.5\\ {}\ 0\ (survival)& if\ {\hat{t}}_i<0.5\end{array}\right.$$

## Results

### Dataset

A real patient Portuguese dataset was provided by the CHUC Cardiology ICU to validate the global assessment framework. It comprises *N*=1544 patients (Table 2) admitted between 2009 and 2016 at the CHUC with all ACS diagnoses (STEMI, NSTEMI and UA).

**Table 2:** Dataset baseline characteristics

Variable	Survival		Death	
	Mean *N*=1319	IQR	Mean *N*=150	IQR
Age	66.87	57–77	77.49	74–83
Systolic blood pressure*	135.05	119.50–150.00	123.07	104.25–140.75
Cardiac frequency*	75.73	64–85	84.02	70–90
Troponin*	41.54	1.03–39.50	60.46	3.78–48.80
Maximum creatinine	112.28	78–111.6	194.77	96–207.75
STEMI*	0.36		0.46	
Maximum Killip	1.35		2.53	

*IQR* interquartile range; *values obtained at patient admission

This validation study was developed with the prior approval of CHCU. The patient data was anonymized.

### GRACE

Table 3 includes the metrics for the quantitative evaluation of trust, according the three identified perspectives: (i) model robustness through stability computation; ii) Confidence based on 95% CI of *G*_*mean*_ ; iii) interpretability based on the correlation of features ranks.

**Table 3:** Trust evaluation (GRACE)

	Stability [−1,1]	G_mean_ 95% CI	Spearman correlation [−1,1]
GRACE	0.506 ± 0.006	[68.2% , 76.6%]	1

The Spearman correlation coefficient is 1, as the GRACE score was adopted as the reference. In fact, the objective of this work is the comparison of ML models with GRACE.

The features’ rank by importance, determined by Shapley values, when applying the GRACE score to this dataset is presented in Table 4.

**Table 4:** GRACE risk factor rank

Features: GRACE rank							
Age	STEMI	Killip class	SBP	Creatinine	Heart rate	Troponin	Cardiac arrest
1	2	3	4	5	6	7	8

Figure 2 presents the SHAP force plot that provides different information: (i) base value that represents the GRACE average prediction (147); (ii) the predicted value for a given patient (105); and (iii) individual variables’ effects. It is possible to confirm that troponin and heart rate values are forces that increase the predicted value, i.e., increase the risk for this specific patient. Contrarily, the values of variables SBP, STEMI, Killip, Age, and Cardiac Arrest are forces that decrease the predicted value in this patient. Although useful, that analysis is limited to individual patients.

**Fig. 6:**
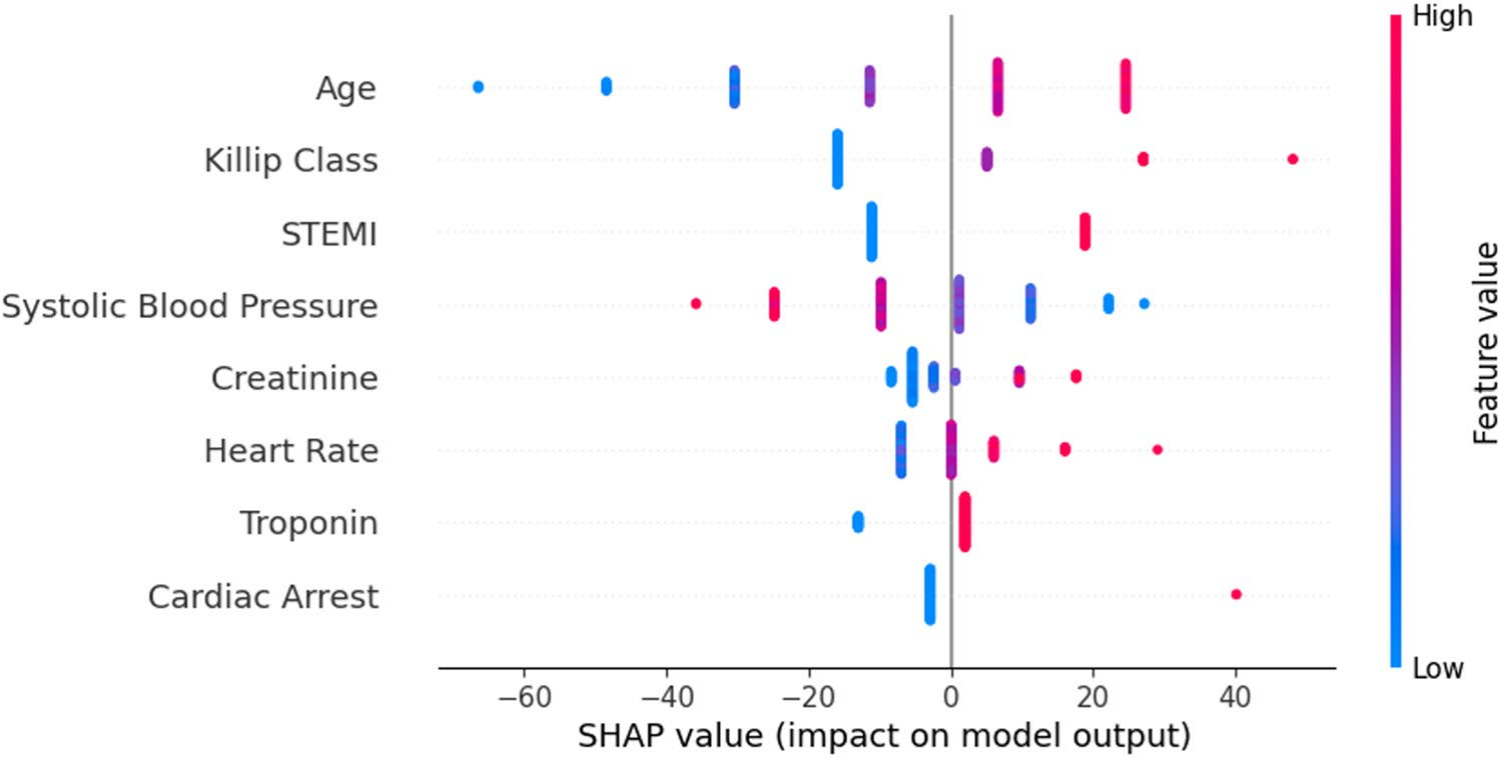
Summary plot (GRACE score)

Figure 6 presents the summary plot, which aggregates the feature importance as well as the respective global impacts in the risk prediction.

It is possible to conclude that higher values on Age, Killip Class, Creatinine, and Heart rate favors the increase of the prediction (patient’s risk). On the contrary, lower values of systolic blood pressure have a similar effect. In fact, it is the only risk factor with this result. For the binary variables, a value of 1 contributes to increase the prediction, and a value of 0 has the opposite effect. These conclusions are in accordance with the points attributed by the GRACE risk score.

In relation to performance assessment, GRACE score is completely predefined, so it does not have training phase. In order to allow the direct comparison with the ML models, i.e., based on the same conditions, GRACE was applied only to the test data (Table 5).

**Table 5:** Performance (GRACE)

	G_mean_	SE	SP
GRACE	72.11 ± 0.07	84.02 ± 0.07	62.1 + 0.00

### Machine learning models

Four ML white-box models were implemented (Fig. 2) in order to be compared with the GRACE score. ML models were developed based on Python scikit-learn and were optimized through a grid search procedure. The reported performance results were obtained based on the mean values of *G*_*mean*_ after 10 runs.

#### Logistic regression

Table 6 comprises the trust metrics of the logistic regression model.

**Table 6:** Trust metrics (LR model)

	Stability [−1,1]	G_mean_ 95% CI	Spearman correlation [−1,1]
Logistic regression	0.634 ± 0.015	[67.3 % , 79.6%]	0.66

**Fig. 7:**
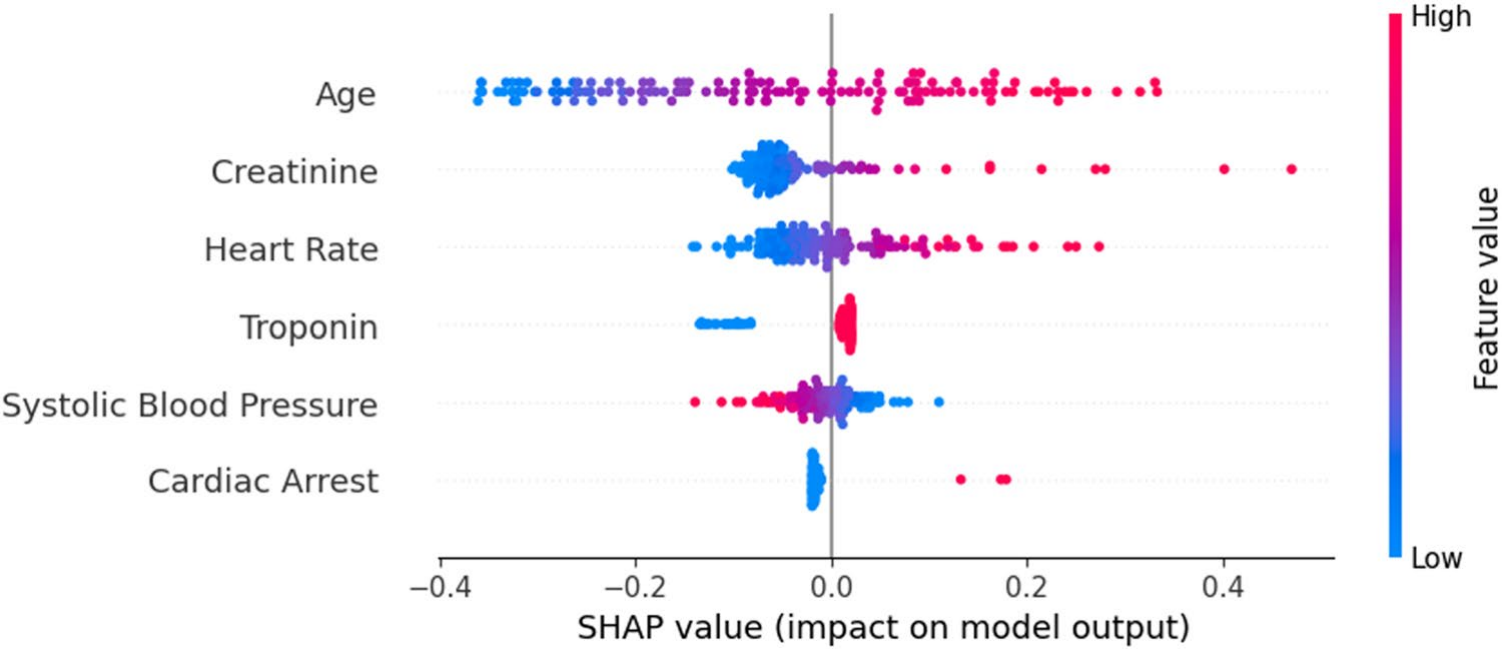
Summary plot (LR model)

The analysis of feature importance, based on Shapley values, allows the identification of Age as the most important feature, followed by creatinine, heart rate, and systolic blood pressure (Fig. 7).

Similarly to GRACE, Age is the feature with more relevance. Higher values on Age, Creatinine, and Heart rate contribute to the increase of the prediction. As expected, lower values of systolic blood pressure also increase the prediction value. The remaining features have a negligible effect.

Table 7 presents the logistic regression model’s performance.

**Table 7:** LR performance

	Gmean %	SE %	SP %
Train	76.95 ± 0.26	74.06 ± 0.48	79.97 ± 0.15
Test	73.57 ± 0.41	68.15 ± 0.41	79.96 ± 0.39

The presented values are the mean and standard deviation based on values of 10 runs

#### Naïve bayes

Table 8 presents the metrics that allow the trust quantification.

**Table 8:** Trust metrics (Naïve Bayes model)

	Stability [−1,1]	G_mean_ 95% CI	Spearman correlation [−1,1]
Naïve Bayes	0.606 ± 0.008	[67.9 % , 79.2%]	0.26

It is important to underline the low value of correlation (feature importance) obtained by this model. Actually, when compared with GRACE, the analysis based on Shapley values returned a very different features’ importance rank.

**Fig. 8:**
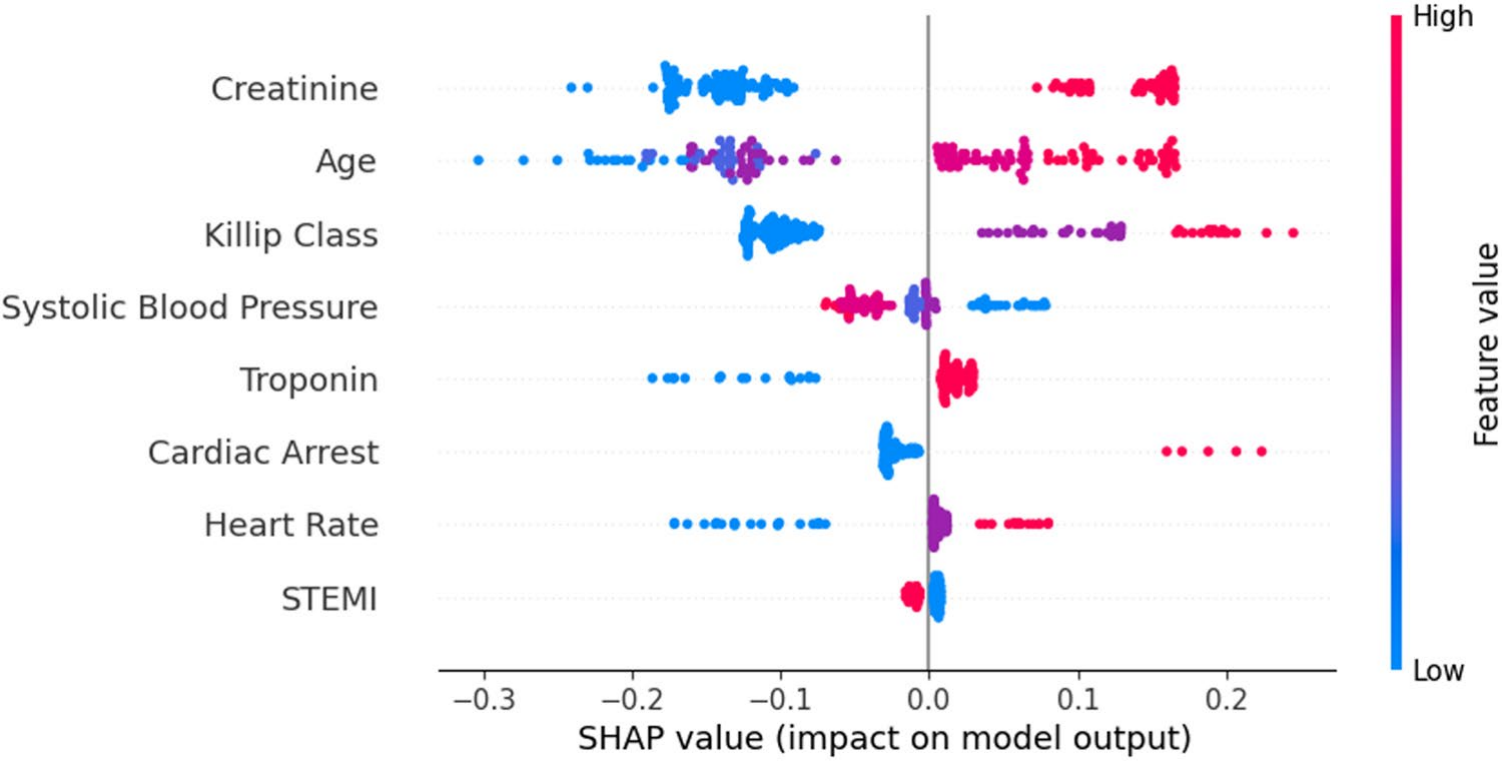
Summary plot (Naïve Bayes model)

Based on Fig. 8, it is possible to confirm that, contrarily to GRACE and LR model, the variable Age is less relevant than Creatinine. High values of creatinine, age, and heart rate contribute to the risk of death. Similarly to the previous models, lower values of SBP contribute to enhance the risk of death.

The discretization of the numerical features is a critical step in the performance of Naïve Bayes classifier [24]; here, the different categories were defined based on the respective clinical significance. Table 9 identifies the different categories of the several features. This discretization is required in order to learn the parameters (conditional probabilities table) of the Bayesian classifier directly from data.

**Table 9:** Categories for the discretization of numerical variables

Variable	Range	Score	Variable	Range	Score	
Age (years)	1 2 3 4 5 6	≤ 40 [40,50] [50,60] [60,70] [70,80] ≥80	SBP (mmHg)	1 2 3 4 5	<120 [120,130] [130,140] [140,180] >180	
Heart rate (bpm)	1 2 3	<60 [60,100] >100	Creatinine (μmol/L)	1 2 3	Men	Women
					<61.9	<53.0
					[61.9,114.9]	[53.0,97.2]
					>114.9	>97.2

The conditional probability table directly derived from the dataset provides the distribution of each category per class (variable Age, Table 10). In categories from 1 to 4 (*Age* < 70 *years*), the conditional probability for each category given survival (class 0) is higher than the probability for each category given death (class 1). Regarding categories 5 and 6 (*Age* ≥ 70 *years*), the probability of each category given death is higher. The probabilities for each category increase as the age of the patient growths. These results are in accordance with the GRACE, as this score attributes more points to older ages. A similar analysis was performed for the other variables, reaching the same conclusions, i.e., the values of conditional probabilities are in accordance with the structure of the GRACE score.

**Table 10:** Conditional probabilities (Age variable)

Class	Category					
	1	2	3	4	5	6
Survival (0)	0.02	0.11	0.19	0.23	0.27	0.18
Death(1)	0.00	0.01	0.05	0.11	0.35	0.48

Table 11 presents the Naïve Bayes model’s performance.

**Table 11:** Naïve Bayes performance

	Gmean %	SE %	SP %
Train	75.81±0.20	73.57±0.43	78.13±0.24
Test	74.39±0.33	71.57±0.70	77.94±0.25

The presented values are the average values of 10 runs

#### Decision trees

Table 12 quantifies the trust of decision tree model.

**Table 12:** Trust metrics (DT model)

	Stability [−1,1]	G_mean_ 95% CI	Spearman correlation [−1,1]
Decision tree	0.648 ± 0.029	[59.4 % , 77.1%]	0.66

The visualization of a DT intuitively provides the features importance of the model, i.e., the hierarchy of decisions provides that information. For instance, it is possible to detect that variables STEMI and Cardiac Arrest are not applied in the decision tree as well as Troponin and Killip have a negligible effect. This perspective was complemented with the features’ rank by importance based on Shapley values. This model is mainly based on the effects of only four features, where, similarly to Naïve Bayes, Creatinine is also the most relevant feature.

In order to preserve the interpretability of the decision tree (DT), the parameter “maximum depth” was set to 3. The performance of the model is presented in Table 13.

**Table 13:** DT performance

	Gmean %	SE %	SP %
Train	78.13±0.28	80.68±2.15	76.00±2.24
Test	71.98±1.26	70.81±1.60	73.96±2.15

The presented values are the average values of 10 runs

#### Rule-based approach

A set of decision rules derived from individual variables was applied. When compared with GRACE score, it is possible to conclude that those rules, directly extracted from data, are coherent with clinical evidence. Table 14 comprises the trust metrics of the rules’ based approach.

**Table 14:** Trust metrics (rule-based approach)

	Stability [−1,1]	G_mean_ 95% CI	Spearman correlation [−1,1]
Rule-based model	0.506 ± 0.009	[68.9 % , 80.1%]	0.83

This approach achieved a very high correlation value between its own feature rank and GRACE’s feature rank.

**Fig. 9:**
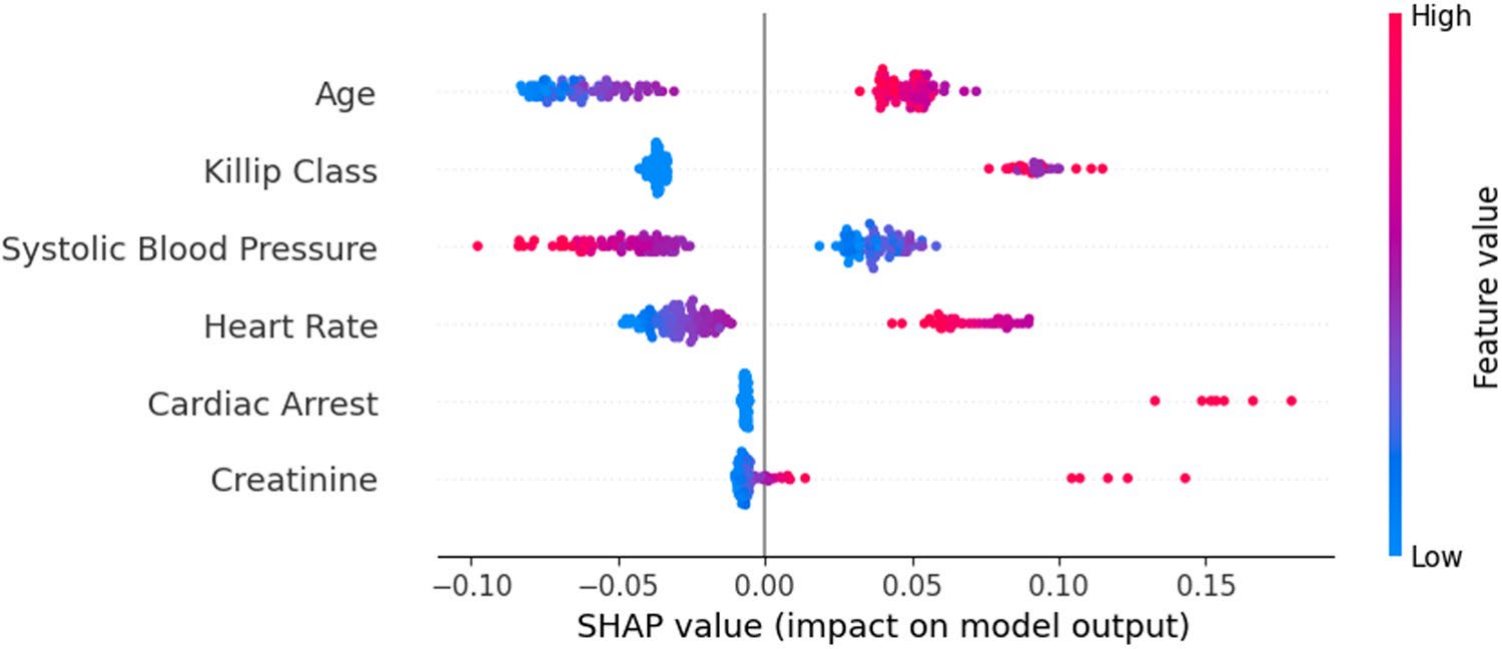
Summary plot (rule-based approach model)

Contrarily to other models, creatinine assumes a less relevant role in this approach (Fig. 9), which contributes to a high correlation with GRACE.

Table 15 comprises the performance values.

**Table 15:** Rule-based approach performance

	Gmean %	SE %	SP %
Train	75.57±0.04	74.34±0.09	76.82±0.03
Test	74.72±0.44	73.36±0.87	76.61±0.15

The presented values are the average values of 10 runs

### Comparison between methods

Table 16 presents the statistic and *p*-value of the Mann-Whitney tests, applied to compare the *G*_*mean*_ for each pair methods (GRACE and machine learning models).

**Table 16:** Mann-Whitney *U* test results

Method 1	Method 2	Test statistic	*p*-value
GRACE	Logistic regression	29.0	1.21^-1^
GRACE	Decision tree	90.0	2.82^-3^
GRACE	Naïve Bayes	0.0	1.83^-4^
GRACE	Rule-based approach	0.0	1.83^-4^
Logistic regression	Decision tree	97.0	4.40^-4^
Logistic regression	Naïve Bayes	10.0	2.83^-3^
Logistic regression	Rule-based approach	0.0	1.83^-4^
Decision tree	Naïve Bayes	0.0	1.83^-4^
Decision tree	Rule-based approach	0.0	1.83^-4^
Naïve Bayes	Rule-based approach	12.0	4.59^-3^

For all tests between pairs of methods, except for the GRACE and logistic regression, it is possible to reject the null hypothesis since the *p*-value<0.05 and therefore conclude that there is a significant difference between the methods results.

**Fig. 10:**
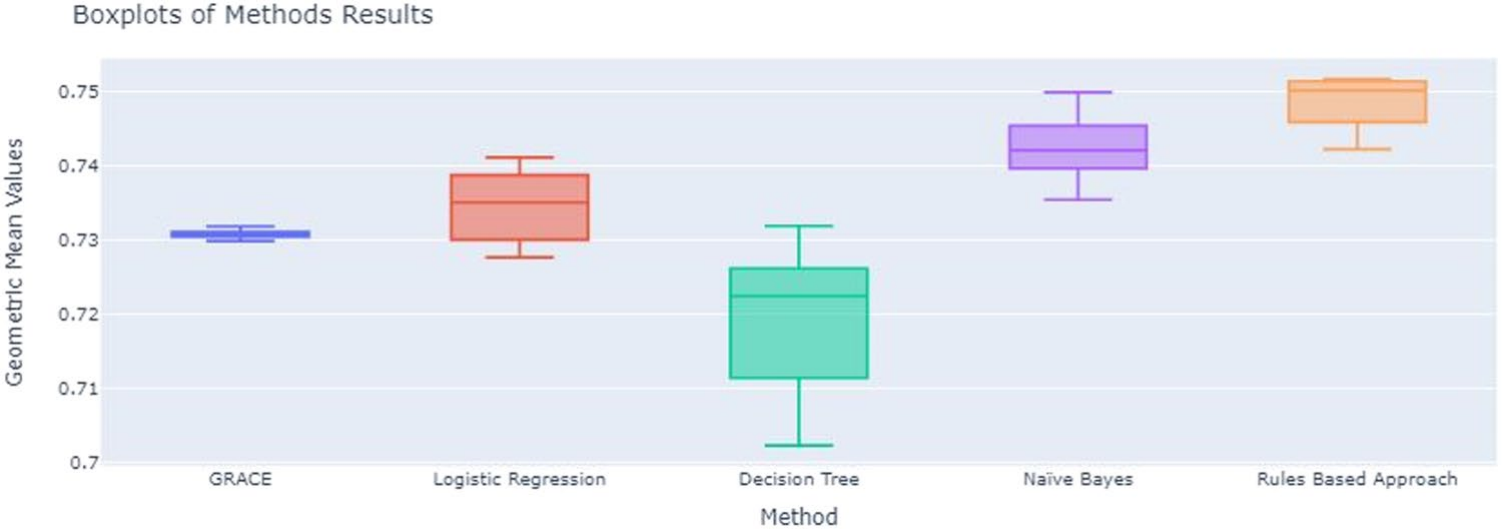
Boxplots of the geometric mean results across the different methods

Figure 10 presents the boxplots of the geometric mean results across the different methods over 10 runs. It is clear that GRACE method has the lowest variability in the results while the decision tree presents the highest. The results between the GRACE and logistic regression seem similar which reinforces the result of the statistical test presented above for these two methods. The decision tree model is the method with the worst results, and the Naïve Bayes as well as the rule-based approach are the methods with the best results. The rule-based approach presents the highest median value for *G*_*mean*_.

## Discussion

Tables 17 and 18 present the aggregated view of trust and performance assessment:

**Table 17:** Trust evaluation

Model	Stability [−1,1]	G_mean_ 95% CI	Spearman corr. [−1,1]
GRACE	0.506	[68.2 % , 76.6 %] (8.4%)	1
Logistic regression	0.634	[67.3 % , 79.6 %] (12.3%)	0.66
Naive Bayes	0.606	[67.9 % , 79.2 %] (11.3%)	0.26
Decision tree	0.648	[59.4 % , 77.1 %] (17.7%)	0.66
Rule-based approach	0.506	[68.9 % , 80.1 %] (11.2%)	0.83

**Table 18:** Performance evaluation (test set)

Model	G_mean_ (%)	SE (%)	SE (%)
GRACE	72.11	84.02	62.10
Logistic regression	73.57	68.15	79.96
Naive Bayes	74.39	71.57	77.94
Decision tree	71.98	70.81	73.96
Rule-based approach	74.72	73.36	76.61

Naïve Bayes and rule-based approach achieved the highest *G*_*mean*_, outperforming the GRACE score (clinical reference), as can be confirmed in Fig. 10. However, rule-based approach achieved higher sensitivity (73.36±0.87) than the NB model (71.57±0.70). This is a relevant issue, as in the clinical practice context, missing a patient with the expected outcome is often more important than an incorrect classification of a patient without the outcome. Thus, false negative errors are usually more important than false positive errors [22]. When compared with GRACE, the rule-based approach presents lower sensitivity but higher geometric mean, which indicates a more balanced performance.

Stability should not be considered isolated, as it can be misleading. It has to be evaluated together with the geometric mean CI. The decision tree model is the one with greater stability (0.648 ± 0.029); however, it is also the one with the highest CI (17.7%), which is a serious drawback and confirms the expected unstable behavior of DT. Moreover, the geometric value of DT is the lowest (59.4%) of all the developed models. On the contrary, the GRACE score has the narrower *G*_*mean*_ confidence interval (8.4%), but it is also one of the models with worst stability (0.506).

These results indicate that the best trade-off between the stability measure and the *G*_*mean*_ confidence interval must be found. NB is the ML model which allows for a better compromise with a stability of 0.606 ± 0.008 and a relatively narrow *G*_*mean*_ CI (11.3%). However, the NB model presents the lowest correlation value (0.26) with GRACE features rank. Contrarily, the rule-based approach has the highest correlation (0.83). The age of the patient is the most important feature in both models (GRACE; rule-based approach). In summary, it is possible to state that rule-based approach is the model that offers the best compromise, when considered the three metrics that quantify trust. It has the best correlation with GRACE features’ rank (interpretability), presenting a similar stability (model robustness) than GRACE (0.506) and a slight undesirable increase of the geometric mean CI (confidence).

## Conclusions

In critical areas, i.e., decisions with significant user impact, ML models need to induce trust in their potential users. Thus, the adoption in effective support to decision depends on trust and performance, but this assessment is not a straightforward concept.

This work proposed an approach to quantify trust considering three different metrics: (i) model robustness (stability); (ii) confidence (*G*_*mean*_ 95% CI); and (iii) interpretability (correlation of the features’ importance rank). Additionally, performance assessment was also carried out.

The validation was accomplished in cardiovascular risk assessment context, namely in the prediction of 6-month mortality risk of ACS patients after hospital admission. Four white-box models were implemented and compared with GRACE score (clinical reference).

The results suggest the potential of this work to combine the quantification of trust, a quite complex concept, and performance. This conjugation is critical to enhance the potential of application of a ML model in the daily clinical practice. It is important to underline that the proposed assessment approach, which is the innovative contribution of this work, can be potentially applied to different critical areas.

As mentioned, this work addresses simultaneously the assessment of trust and performance of ML models. Regarding performance perspective, despite the potential of the proposed strategy, the validation process should be reinforced based on other datasets. In parallel, the assessment of the performance of an individual instance would be very important to increase trust. Actually, even if the model is, on average, very accurate, individual predictions may be more or less reliable. This individual reliability assessment is a line of research that is being pursued by this research team.

## References

[1] Margot E. Kaminski (2019) The right to explanation, Explained, 34 Berkeley Tech. L.J. 189. https://scholar.law.colorado.edu/faculty-articles/1227

[2] Linardatos P et. al. (2020) Explainable AI: a review of machine learning interpretability methods. Entropy 23, 1. 10.3390/e23010018 10.3390/e23010018 PMC7824368 33375658

[3] Abedin B et al (2022) Managing the tension between opposing effects of explainability of artificial intelligence: a contingency theory perspective. Internet Research 3(32):425–453. 10.1108/INTR-05-2020-0300

[4] Mashrur A et al (2020) Machine learning for financial risk management: a survey. IEEE Access 8:203203–203223. 10.1109/ACCESS.2020.3036322

[5] Valente F, Henriques J, Paredes S et al (2021) A new approach for interpretability and reliability in clinical risk prediction: acute coronary syndrome scenario. Artif Intell Med 7:102113. 10.1016/j.artmed.2021.102113 10.1016/j.artmed.2021.102113 34127242

[6] WHO (2022) Cardiovascular diseases (CVDs). https://www.who.int/news-room/fact-sheets/detail/cardiovascular-diseases-(cvds). accessed July 2023.

[7] Araújo P et al (2005) TIMI, PURSUIT, and GRACE risk scores: sustained prognostic value and interaction with revascularization in NSTE-ACS. Eur Heart J 26:865–872. 10.1093/eurheartj/ehi187 15764619 10.1093/eurheartj/ehi187

[8] Carvalho DV et al (2019) Machine learning interpretability: a survey on methods and metrics. Electronics 8(8):832. 10.3390/electronics8080832

[9] Carrington A, Fieguth P, Chen H (2018) Measures of model interpretability for model selection. In: 2nd International Cross-Domain Conference for Machine Learning and Knowledge Extraction (CDMAKE), Aug 2018, Hamburg, Germany, pp 329–349. 10.1007/978-3-319-99740-7_24

[10] Charlton C et al (2023) Development of prediction models for one-year brain tumour survival using machine learning: a comparison of accuracy and interpretability. Comput Methods Programs Biomed 233:107482. 10.1016/j.cmpb.2023.107482 36947980 10.1016/j.cmpb.2023.107482

[11] Arya V et al. (2021) One explanation does not fit all: a toolkit and taxonomy of AI explainability techniques, Informs. https://research.ibm.com/publications/one-explanation-does-not-fit-all-a-toolkit-and-taxonomy-of-ai-explainability-techniques

[12] Doshi-Velez F, Kim B (2017) Towards a rigorous science of interpretable machine learning. arXiv: Machine Learning. 10.48550/arXiv.1702.08608

[13] Murdoch J (2019) Definitions, methods, and applications in interpretable machine learning. Physical Sciences, PNA. https://www.pnas.org/doi/full/10.1073/pnas.1900654116 10.1073/pnas.1900654116 PMC6825274 31619572

[14] Waa J et al (2020) Interpretable confidence measures for decision support systems. Int J Hum-Comp Stud 144:102493. 10.1016/j.ijhcs.2020.102493

[15] Burkart N, Huber M (2021) A survey on the explainability of supervised machine learning. J Artif Intell Res 70:245–317

[16] Molnar C, Casalicchio G, Bischl B (2020) Interpretable machine learning – a brief history, state-of-the-art and challenges. In: Koprinska I et al (eds) ECML PKDD 2020 Workshops. ECML PKDD 2020. Communications in Computer and Information Science, vol 1323. Springer, Cham. 10.1007/978-3-030-65965-3_28

[17] Ahmad M, Eckert C, Teredesai A (2018) Interpretable machine learning in healthcare, Proceedings of the 2018 ACM international conference on bioinformatics, computational biology, and health informatics. 10.1145/3233547.3233667

[18] Lundberg S, Lee S (2017) A unified approach to interpreting model predictions. In: Advances in Neural Information Processing Systems 30 (NIPS)

[19] Anderson P et al (2003) Understanding logistic regression analysis in clinical reports: an introduction. Ann Thorac Surg 75:753–757. 10.1016/s0003-4975(02)04683-0 12645688 10.1016/s0003-4975(02)04683-0

[20] Paredes S et al (2011) Long term cardiovascular risk models’ combination. Comput Methods Programs Biomed 101(3):231–242. 10.1016/j.cmpb.2010.12.015 21255861 10.1016/j.cmpb.2010.12.015

[21] Roseiro M, Henriques J, Paredes S et al (2023) An interpretable machine learning approach to estimate the influence of inflammation biomarkers on cardiovascular risk assessment. Comput Methods Programs Biomed 230:107347. 10.1016/j.cmpb.2023.107347 36645940 10.1016/j.cmpb.2023.107347

[22] Steyerberg W (2009) Clinical prediction models – a practical approach to development. In: Validation and Updating. ISBN: 978-0-387-77243-1, Statistics for Biology and Health. Springer

